# Freshwater Clam Extract Mitigates Neuroinflammation and Amplifies Neurotrophic Activity of Glia: Insights from In Vitro Model of Neurodegenerative Pathomechanism

**DOI:** 10.3390/jcm11030553

**Published:** 2022-01-22

**Authors:** Muh-Shi Lin, Shu-Mei Chen, Kuo-Feng Hua, Wei-Jung Chen, Cho-Chen Hsieh, Chai-Ching Lin

**Affiliations:** 1 Division of Neurosurgery, Department of Surgery, Kuang Tien General Hospital, Taichung 43303, Taiwan; neurosurgery2005@yahoo.com.tw; 2 Department of Biotechnology and Animal Science, College of Bioresources, National Ilan University, Yilan 26047, Taiwan; kuofenghua@gmail.com (K.-F.H.); wjchen@niu.edu.tw (W.-J.C.); q2862540@gmail.com (C.-C.H.); 3 Department of Biotechnology, College of Medical and Health Care, Hung Kuang University, Taichung 43302, Taiwan; 4 Department of Health Business Administration, College of Medical and Health Care, Hung Kuang University, Taichung 43302, Taiwan; 5 Department of Neurosurgery, Taipei Medical University Hospital, Taipei Medical University, Taipei 110, Taiwan; nschenis@gmail.com; 6 Department of Surgery, School of Medicine, Taipei Medical University, Taipei 110, Taiwan

**Keywords:** freshwater clam extract (FCE), neuroinflammation, oxidative stress, neurotrophic factor, primary glial cell culture

## Abstract

Background. An extensive body of research suggests that brain inflammation and oxidative stress are the underlying causes of Parkinson’s disease (PD), for which no potent therapeutic approach exists to mitigate the degradation of dopamine neurons. Freshwater clams, an ancient health food of Chinese origin, have been documented to exhibit anti-inflammatory and antioxidant effects. We previously reported that freshwater clam extract (FCE) can attenuate astrocytic activation and subsequent proinflammatory cytokine production from substantia nigra in an MPTP-induced PD mouse model. This article provides insight into the potential mechanisms through which FCE regulates neuroinflammation in a glia model of injury. Materials and methods. In total, 1 μg/mL lipopolysaccharide (LPS) and 200 μM rotenone were conducted in primary glial cell cultures to mimic the respective neuroinflammation and oxidative stress during injury-induced glial cell reactivation, which is relevant to the pathological process of PD. Results. FCE markedly reduced LPS-induced neuroinflammation by suppressing NO and TNF-α production and the expression of pro-inflammatory cytokines. In addition, FCE was effective at reducing rotenone-induced toxicity by diminishing ROS production, promoting antioxidant enzymes (SOD, catalase, and GPx) and minimizing the decline in glial-cell-secreted neurotrophic factors (GDNF, BDNF). These impacts ultimately led to a decrease in glial apoptosis. Conclusions. Evidence reveals that FCE is capable of stabilizing reactive glia, as demonstrated by reduced neuroinflammation, oxidative stress, the increased release of neurotrophic factors and the inhibition of apoptosis, which provides therapeutic insight into neurodegenerative diseases, including PD.

## 1. Introduction 

Astrocytes are the most abundant glial cells for sustaining neuronal function in the human brain. Crosstalk between astrocytes and neurons is crucial to maintain homeostasis because glial cells provide architectural support and nutritional supply for neurons in the central nervous system (CNS). Specifically, astrocyte-mediated neuroprotection has been proposed to be due to the limiting of neuronal death from excitotoxins and oxidants [1,2], maintenance energy metabolism, and regulating osmolarity for volume regulation [3]. Furthermore, glial cell-line-derived neurotrophic factor (GDNF) and brain-derived neurotrophic factor (BDNF) secreted from glia, including astrocytes and microglia, have been shown to protect neurons against apoptosis. GDNF was reported to enhance survival for injured nigrostriatal dopaminergic neurons in an in vitro model of Parkinson’s disease (PD) [4,5]. 

Emerging evidence has suggested that excessive inflammation, mitochondrial dysfunction, increased oxidative stress, excitotoxicity, ubiquitin-proteasome system dysfunction, alpha-synuclein aggregation, and Lewy body formation may underlie the pathogenesis of PD [6,7,8]. Among these pathogeneses, neuroinflammation plays a critical role in the progression of PD and Alzheimer’s disease (AD) [9,10,11]. To maintain CNS function, glial cells can be activated and act as immunocompetent cells to protect the host from CNS diseases, including PD. Activated glial cells, consisting of microglia or astrocytes, promote the release of pro-inflammatory cytokines, reactive oxygen species (ROS), nitric oxide (NO), glutamate, phospholipases, and proteases, which, through the release of these neurotoxic mediators, ultimately contribute to the progressive neuronal damage observed in PD [12,13,14]. Consequently, impaired mitochondria-specific autophagy, accompanied by excessive inflammation, which is linked to a number of genes implicated in PD, leads to mitochondrial dysfunction and potentially evokes direct neuronal dysfunction and neurodegeneration [15]. Taken together, glial cells function in both beneficial and detrimental roles as part of the pathophysiological mechanisms of PD. As such, any novel approach that would enhance neuroprotection and reduce the inflammatory activation of glial cells would be an essential therapeutic advance.

As widely popular edible shellfish in Asia, freshwater clams contain high levels of lipophilic phytosterols [16]. A number of in vitro and in vivo studies have found that freshwater clams exert many medical and biological effects, including cholesterol reduction [17] and hepatoprotection [18]. Previously, we demonstrated that freshwater clam extract (FCE) exerted anti-inflammatory effects in vivo and in vitro, and exhibited therapeutic potential in the treatment of neurodegenerative diseases. Concretely, in an MPTP-induced mouse PD model, FCE stabilized dopamine neurons in the substantia nigra pars compacta (SNpc) through the stimulation of neurotrophic factors, the reduction of astrocyte activation and the inhibition of injury-induced neuronal depletion [19]. Furthermore, FCE can downregulate ERK1/2, JNK1/2, p38 phosphorylation and NF-κB activity, thereby reducing NO, IL-1β, IL-6 and TNF-α in lipopolysaccharide (LPS)-activated macrophages [20]. 

With the aim of achieving better insight into the pathological mechanisms involved in PD generation, we further explored the detailed mechanisms implicated in neurodegenerative diseases by employing an in vitro model of glial cell activation and mitochondrial dysregulation induced by LPS and rotenone, as recognized triggers of neuroinflammation [21,22] and PD pathogenesis [23], respectively. As such, in the present study, we attempted to estimate: (1) the inhibitory effect of FCE on injury-reactivated proinflammatory reaction and oxidative stress and (2) the potentiating effect upon the secretion of neurotrophic factors in glial cells. 

## 2. Materials and Methods

### 2.1. Preparation of Freshwater Clam Extract (FCE)

FCE was prepared by the method described in previous papers [20]. Freshwater clams, *Corbicula fluminea*, were provided by the LiChuan Aquaculture Farm (Hualien, Taiwan). The freshwater claim powder of the edible portion of C. flluminea (50 g, dry weight) was extracted with 500 mL 99.9% ethanol (32221, Sigma, Sigma-Aldrich, St. Louis, MO, USA) for 16 h. The extract was concentrated and dried to produce ethanol (ECF). ECF (20 g) was sub-partitioned with 300 mL ethyl acetate (270989, Sigma, Sigma-Aldrich, St. Louis, MO, USA). The collected sub-fraction was dried and stored at 4 °C. The amount of FCE was expressed in terms of dry weight.

### 2.2. Preparation of Primary Glial Cell Cultures

Primary glial cell cultures were prepared completely in accordance with the published protocols [24]. As a result, cultures of microglia, astrocytes, and oligodendrocytes were prepared to mimic the microenvironment of the real brain, in which glial cells are collectively, rather than individually, implicated in the pathogenesis of neurodegenerative diseases. Briefly, P0–P2 neonatal mouse pups were decapitated using sterile large scissors. The heads were gently placed into a petri dish. Forceps were used to hold the nose portion of the head, while the skin was cut by following the midline. The entire brain was exposed while keeping the olfactory bulbs and brain tissue intact. The brain was then placed into a new petri dish containing cold DMEM. Under a dissecting magnifying glass, the brainstem and cerebellum were removed. The olfactory bulbs were clamped to remove the meningeal layer from both hemispheres. The hemispheres were carefully opened and the choroidal plexus covering on the inside removed. The forebrains were transferred to a Falcon tube containing cold DMEM. The nervous tissue was triturated and dissociated with a serum-coated Pasteur pipette. The tubes were then centrifuged for 15 min to 200× *g*. The supernatant was then discarded and the pellet suspended in 25 mL of warm DMEM with 10% fetal bovine serum and 10% horse serum per six forebrains. This mixture was then seeded into Poly-D-Lysine-covered 75 cm^2^ flasks and incubated at 37 °C in 5% carbon dioxide for 7 to 10 days, without changing the culture medium during this time. 

### 2.3. In Vitro Models for Injury-Induced Neurodegeration 

Rotenone is known to produce ROS by inhibiting mitochondrial complex I activity [25] and triggering inflammatory effects [26]. Rotenone has been shown to readily cross the blood–brain barrier and accumulate throughout the brain, impairing mitochondrial function, ultimately leading to neurodegeneration [25,27]. Rotenone administration has been shown to directly increase ROS production and contribute to dopaminergic neuronal injury [25,28]. In this study we used a glial cell culture model and rotenone was administrated to induce inflammation and ROS production with the aim of mimicking the situation commonly encountered in PD.

### 2.4. Cell Viability Analysis

Cell viability was determined using an MTT assay. The MTT assay provided a sensitive measurement of the metabolic status cell, particularly the status of the mitochondria, which may reflect early redox change. Primary glial cells were seeded in 96 well plates at a density of 1 × 10^4^ cell/well and incubated for 24 h prior to the experimental treatments. The cells were then subjected to the required treatments. After 24 h incubation, MTT (0.5 mg/mL) was added to each well. Following an additional 1 h incubation at 37 °C, the supernatant was then removed. One hundred μL of DMSO was added to dissolve the formazan crystals. The absorbance was measured at 450 nm using a Bio-Rad iMark™ microplate reader (Bio-Rad Laboratories, Inc. Hercules, CA, USA). Wells without cells were used as blanks and were subtracted as background from each sample. Results were expressed as a percentage of control.

### 2.5. Measurement of Intracellular ROS

ROS production was measured using an oxidation-sensitive fluorescent probe 2′7′-dichlorofluorescein diacetate (DCF-DA, D6883, Sigma, Sigma-Aldrich, St. Louis, MO, USA) method, which is based on the ROS-dependent oxidation of DCF-DA to the highly fluorescent compound dichlorofluorescein (DCF). Primary glial cells (5 × 10^5^ cells/well) were seeded in a 6 well plate for 24 h and then exposed to the vehicle, rotenone (200 μM), or rotenone co-treatment FCE (250 μg/mL, 500 μg/mL). After incubation for 24 h, the medium was removed and the cells were washed twice with PBS. After washing, the cells were incubated with DCF-DA (10 μM) for 30 min at 37 °C in the dark. The stained cells were detected using an Accuri C6 cytometer (BD Biosciences, USA) and the data were analyzed using CellQuestPro (BD Biosciences, San Diego, CA, USA).

### 2.6. Flow Cytometric Analysis of Cell Cycle Distribution

Primary glial cells (5 × 10^5^ cells/well) were seeded in a 6 well plate for 24 h and then exposed to the vehicle, rotenone (200 μM), or rotenone co-treatment FCE (250 μg/mL, 500 μg/mL) to analyze the cell cycle phase distribution. After incubation for 24 h, the cells were washed twice with PBS and then fixed 24 h in 70% ethanol at −20 °C. The fixed cells were then washed twice with PBS and 10 mg/mL RNase A (R6513, Sigma, Sigma-Aldrich, St. Louis, MO, USA) was added. Propidium iodide (P4170, Sigma, Sigma-Aldrich, St. Louis, MO, USA) was then added to the tubes at a final concentration of 0.05 mg/mL. The samples were then incubated at 4 °C for 30 min in the dark. The cell cycle was determined using an Accuri C6 cytometer (BD Biosciences, San Diego, CA, USA) and the data were analyzed using CellQuestPro (BD Biosciences, San Diego, CA, USA).

### 2.7. Flow Cytometry Analysis of Cell Apoptosis (Annexin V/Propidium Iodide Assay)

An apoptosis assay was performed using Annexin V/FITC Kit (BD Biosciences, San Diego, CA, USA), following the manufacturer’s protocol; primary glial cells (5 × 10^5^ cells/well) were seeded in a 6 well plate for 24 h and then exposed to the vehicle, rotenone (200 μM), or rotenone co-treatment FCE (250 μg/mL, 500 μg/mL). After incubation for 24 h, the cells were collected and washed twice with PBS. The cells were diluted to 10^6^ cells/mL in Annexin V binding buffer and stained with fluorescein isothiocyanate Annexin V and propidium iodide. The stained cells were detected using an Accuri C6 cytometer (BD Biosciences, San Diego, CA, USA) and the data were analyzed using CellQuestPro (BD Biosciences, San Diego, CA, USA).

### 2.8. Nitric Oxide Assay

The quantity of nitrite accumulated in the culture medium was measured as an indicator of NO production. Briefly, primary glial cells (3 × 10^5^ cells/well) were seeded in a 6 well plate for 24 h and then exposed to the vehicle, LPS, or LPS (1 μg/mL) co-treatment FCE (100, 200, 300, 400 μg/mL). After incubation for 24 h, 100 µL of cell culture medium was mixed with 100 µL of Griess reagent (1% sulphanilamide and 0.1% naphthyl ethylenediamine dihydrochloride in 2.5% phosphoric acid). The mixture was incubated at room temperature for 10 min and the absorbance at 550 nm was read using a ELISA reader, Bio-Rad iMark™ microplate reader (Bio-Rad Laboratories, Inc. Hercules, CA, USA). Fresh culture medium was used as a blank in every experiment.

### 2.9. TNF-α Assay

TNF-α were assayed using DuoSet immunoassay kits (DY410, R&D Systems, Minneapolis, MN, USA). The ELISA was performed following the instructions provided by the manufacturer. Briefly, primary glial cells (3 × 10^5^ cells/well) were seeded in a 6 well plate for 24 h and then exposed to the vehicle, LPS (1 μg/mL), or LPS co-treatment FCE (100, 200, 300, 400 μg/mL). After incubation for 24 h, 96 well ELISA microplates were coated with a capture antibody and after blocking, the cytokine samples or standards were added to the coated plates, followed by a biotin-conjugated detection antibody. The antibody binding was detected with an HRP-conjugated Avidin plus a soluble colorimetric substrate. The absorbance was taken at 450 nm using a Bio-Rad iMark™ microplate reader (Bio-Rad Laboratories, Inc., Hercules, CA, USA). The cytokine concentrations were calculated based on absorbance values, cytokine standards and sample dilution factors and expressed as nanograms per milliliter.

### 2.10. Real-Time Quantitative RT-PCR Analysis (qRT-PCR) for Primary Glial Cells

Primary glial cells (5 × 10^5^ cells/well) were seeded in a 6 well plate for 24 h and then exposed to the vehicle, rotenone (200 μM), or rotenone co-treatment FCE (250 μg/mL, 500 μg/mL). After incubation for 24 h, total RNAs from cells were extracted using Trizol (Invitrogen) following the instructions provided by the manufacturer. Briefly, each cell was homogenized in 1 mL of Trizol reagent. After 5 min at room temperature, 200 μL chloroform was added, shaken vigorously for 15 s and incubated for 3 min, centrifuged at 12,000× *g* for 15 min at 4 °C. The upper aqueous phase was then collected and 400 μL isopropanol added, mixed well and then incubated for −20 °C overnight. On the second day, the sample was centrifuged at 12,000× *g* for 10 min at 4 °C; the supernatant was discarded and 1 mL 75% ethanol was added to wash the precipitated RNA. The sample was centrifuged at 7500 × *g* for 5 min at 4 °C, the pellet dried and resuspended in 20 μL DEPC treated water, then preserved at −20 °C. For qRT-PCR, first-strand cDNA was synthesized using PrimeScript RT reagent Kit (RR037A, Takara Bio, Inc., Shiga, Japan) with both oligo (dT) primer and random hexamers. The qRT-PCR was performed as described in the method of SYBR premix Ex Taq (RR420A, Takara Bio). The reaction mixtures were initially heated at 95 °C for 10 min and then subjected to 40 thermal cycles (95 °C for 15 s and 60 °C for 1 min) with a StepOne Real-Time PCR System thermocycler (Applied Biosystems). The subsequent primer is presented in Table 1. Actin mRNA levels were used for normalization. The fold-change for gene expression was calculated using 2^−ΔΔ^Ct.

### 2.11. Statistical Analysis

SigmaStat (version 3.5, 2006, Sigma-Aldrich, St. Louis, MO, USA) was used in the data analysis. Data are shown as mean ± SD. The significance of differences between the values was determined by Tukey post hoc test after evaluating differences among treatment groups by one-way ANOVA; *p* < 0.05, *p* < 0.01, and *p* < 0.001 were taken as significant.

## 3. Results

### 3.1. Pharmacological Effect of FCE and Rotenone on Cell Survival in Primary Glial Cells

**Figure 1:**
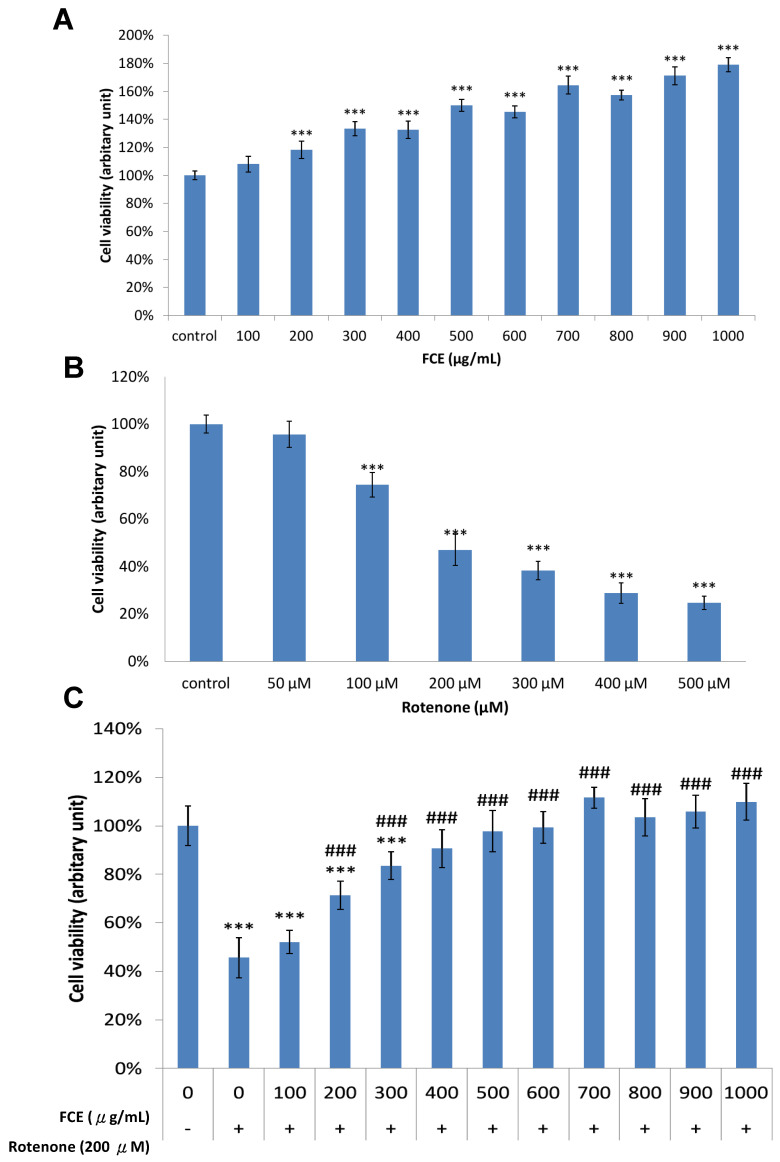
Effect of (**A**) FCE and (**B**) rotenone on cell viability. (**C**) Protective effect of FCE on rotenone-induced cytotoxicity in primary glial cells. Primary glial cells were co-treated with different concentration of FCE with 200 μM rotenone for 24 h. Cell viability was measured by MTT assay and the results were expressed as a percentage of the control absorbance. Data represent the mean ± SD (*n* = 8). Significantly different are *** *p* < 0.001 compared to control; ^###^ *p* < 0.001 compared to cells with 200 μM rotenone.

We first examined whether FCE or rotenone influenced cell survival in mouse primary glial cells. The cells were treated with different concentrations of FCE 100–1000 μg/mL (Figure 1A) or rotenone 50–500 μM (Figure 1B) for 24 h. An MTT assay was conducted to determine the extent of cell survival (Figure 1). FCE enhanced the viability of primary glial cells in a dose-dependent manner. Upon exposure to FCE concentrations up to 200 μg/mL, the viability of the primary glial cells was significantly elevated compared to the control cells (*** *p* < 0.001, compared to control, Figure 1A). As illustrated in Figure 1B, rotenone evoked a dose-dependent cytotoxicity in primary glial cells (*** *p* < 0.001, compared to control). Only 47% of the cells were viable under the presence of 200 μM rotenone, compared to the control cells. Hence, in subsequent experiments, cytotoxicity was elicited with 200 μM of rotenone for 24 h.

### 3.2. FCE Protected against Rotenone-Induced Cytotoxicity in Primary Glial Cells

In the next step, we attempted to determine the effect of FCE on neuronal protection in the MTT reduction assay. The primary glial cells were co-conditioned with 200 μM rotenone and FCE 100–1000 μg/mL for 24 h to determine the protective effect of FCE against rotenone-induced loss of cell viability. As indicated in Figure 1C, compared with control cells untreated with rotenone, the survival rate of the primary glial cells processed with 200 μM rotenone declined noticeably to as low as 46% (*** *p* < 0.001, compared to control). Compared to this, when the rotenone-challenged cells were treated with FCE (100–500 μg/mL), the survival rate significantly increased, from 46% to 71%, 84%, 91% and 98%, respectively (^###^ *p* < 0.001, compared to rotenone). The survival of the rotenone-challenged cells with 500–1000 μg/mL of FCE displayed no significantly detrimental impact as compared to control cells (without rotenone toxicity). These results suggest that FCE protects primary glial cells from the rotenone-induced depletion of cell viability.

### 3.3. FCE Exerted Promising Anti-Inflammatory Effects against Injury-Induced Neuroinflammation in Primary Glial Cells

Under the circumstances of neuroinflammation, pro-inflammatory cytokines such as IL-6, IL-1 β and TNF- α give rise to the overproduction of NO, which accelerates the neurodegenerative process and ultimately facilitates neuronal death [29]. 

**Figure 2:**
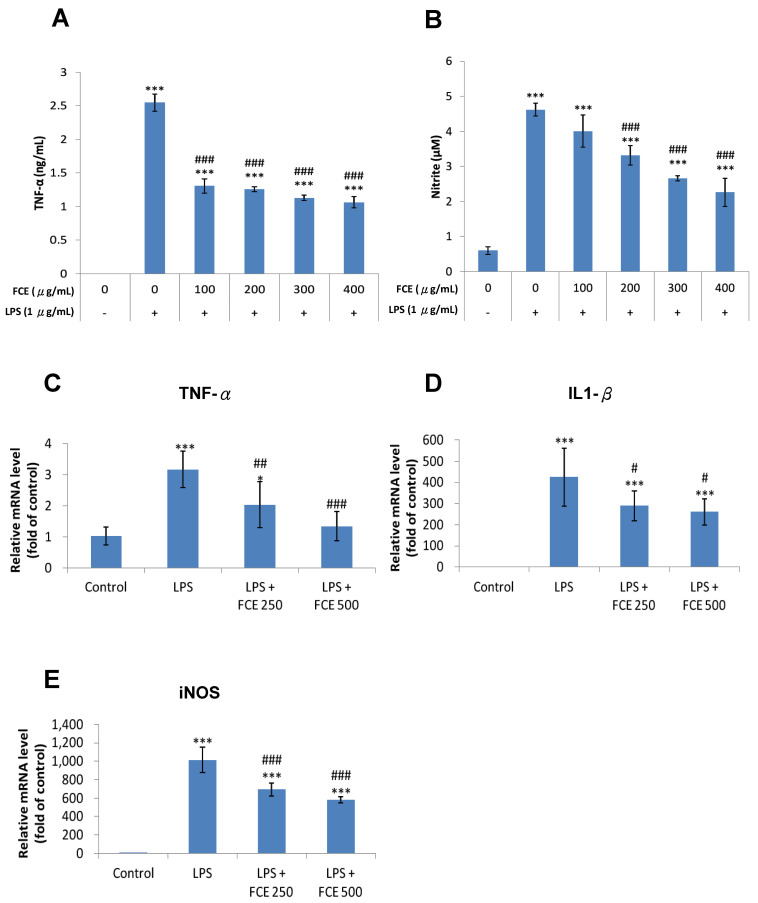
FCE attenuated the production of nitric oxide (NO) and TNF-α, as well as mRNA expression of TNF-α, IL-1β and iNOS in LPS-challenged primary glial cells. (**A**,**B**) Cells were treated with vehicle or LPS 1 μg/mL and FCE (0, 100, 200, 300, 400 μg/mL) for 24 h. (**A**) The effect of FCE on LPS-induced TNF-α production. (**B**) The effect of FCE on LPS-induced NO production. (**A**,**B**) Data represent the mean ± SD (*n* = 8). Significantly different are *** *p* < 0.001 compared to control; ^###^ *p* < 0.001 compared to LPS. (**C**–**E**) Primary glial cells were treated with vehicle or LPS 1 μg/mL and FCE (0, 250, 500 μg/mL) for 24 h. Expression of (**C**) TNF-α, (**D**) IL-1β and (**E**) iNOS mRNA was quantified by real-time quantitative reverse-transcription polymerase chain reaction (qRT-PCR). (C–E) Data represent the mean ± SD (n = 6). Significantly different are * *p* < 0.05, ** *p* < 0.01 and *** *p* < 0.001 compared to control; ^#^ *p* < 0.05, ^##^ *p* < 0.01 and ^###^ *p* < 0.001 compared to LPS.

Under the ELISA assay and Griess reagent system, cytokine levels were analyzed to examine the anti-inflammatory effects of FCE on the pro-inflammatory cytokines TNF-α and NO, respectively, in the LPS-challenged glial cells. A total of 1 μg/mL LPS markedly enhanced TNF-α production by as much as 2.55 ng/mL (*** *p* < 0.001, Figure 2A) compared to the control group. The levels of TNF-α production in the LPS-challenged cells with the FCE treatment (100, 200, 300, and 400 µg/mL) dropped to 1.31 (^###^ *p* < 0.001), 1.26 (^###^ *p* < 0.001), 1.13 (^###^ *p* < 0.001) and 1.06 ng/mL (^###^ *p* < 0.001), respectively, compared to the LPS group without FCE (Figure 2A). 

While the primary glial cells were treated with LPS, NO production was significantly higher, up to 4.62 μM, compared to the control group (*** *p* < 0.001, Figure 2B). Furthermore, the primary glial cells challenged with LPS co-treated with FCE (200, 300, and 400 μg/mL) showed a significant decrease in NO production levels of 3.32 (^###^ *p* < 0.001), 2.66 (^###^ *p* < 0.001), and 2.26 μM (^###^ *p* < 0.001), respectively, compared to the LPS group (Figure 2B).

These results revealed that FCE exhibited a superior inhibitory effect on neuroinflammation in terms of TNF-α and NO production in primary glial cells following LPS challenge. 

### 3.4. FCE Diminished Pro-Inflammatory mRNA Expression of TNF-α, iNOS and IL-1 Triggered by LPS in Primary Glial Cells

A qRT-PCR was then conducted to determine the expression of mRNA. Compared with the control group, the expression levels of mRNA for TNF-α (*** *p* < 0.001, Figure 2C), IL-1β (*** *p* < 0.001, Figure 2D) and iNOS (*** *p* < 0.001, Figure 2E) and were remarkably increased in the primary glial cells with the 1 μg/mL LPS treatment. 

The LPS-stimulated cells processed with FCE manifested reduced mRNA expression levels of TNF-α (FCE 250μg/mL, ^##^ *p* < 0.01; FCE 500 μg/mL, ^###^ *p* < 0.001, Figure 2D), IL-1β (FCE 250 μg/mL, ^#^ *p* < 0.05; FCE 500 μg/mL, ^#^ *p* < 0.05, Figure 2D) and iNOS (FCE 250μg/mL, ^###^ *p* < 0.001; FCE 500 μg/mL, ^###^ *p* < 0.001, Figure 2E), compared to the LPS group without FCE treatment. Results confirmed the anti-inflammatory effects of FCE against LPS-triggered neuroinflammation in primary glial cells. 

### 3.5. FCE Attenuated Rotenone-Induced Oxidative Stress in Primary Glial Cells

There is a significant amount of evidence that ROS are involved in the pathogenesis of various neurodegenerative diseases, including PD [30]. ROS were shown to be produced by rotenone through inhibition of mitochondrial complex I activity [31].

To characterize alterations in intracellular ROS in mouse primary glial cells following rhodopsin-induced cell death and FCE-mediated protection, we measured ROS production in primary glial cells with the fluorescent dye, DCF-DA.

**Figure 3:**
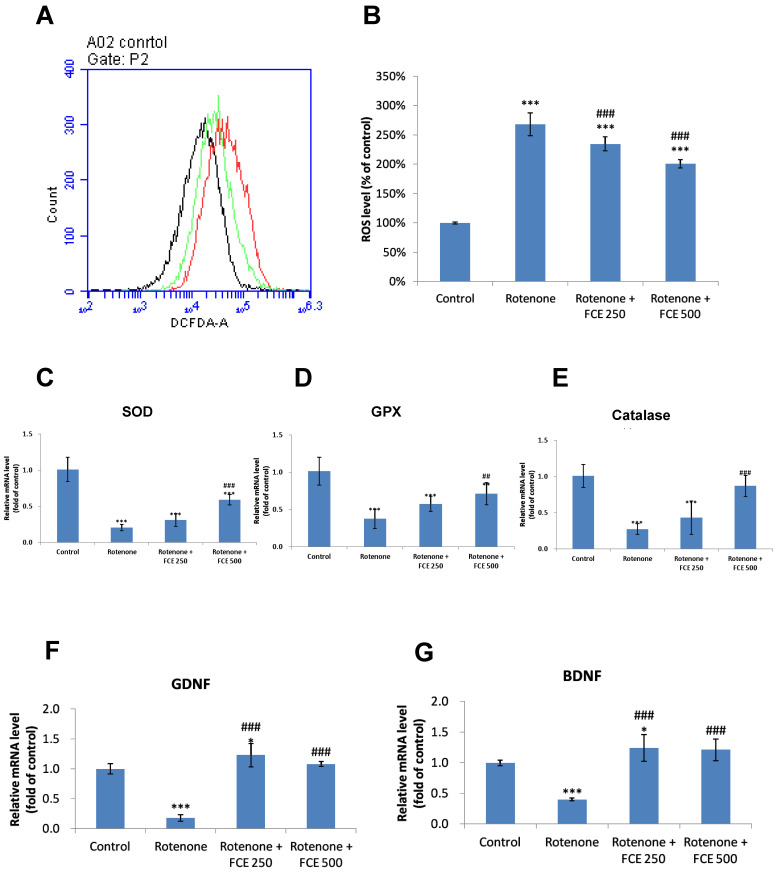
FCE diminished the production of reactive oxygen species (ROS), and enhanced rotenone-attenuated antioxidant enzymes and neurotrophic factors in rotenone-challenged primary glial cells. Primary glial cells were treated with vehicle (rotenone 200 μM) or rotenone + FCE (250, 500 μg/mL) for 24 h. Intracellular ROS accumulation was measured with a fluorescent probe designated DCF-DA. (**A**) Representative histogram of fluorescence from experimental groups. (**B**) Mean fluorescence obtained from the histogram statistics. Data represent the mean ± SD (for **A**,**B**, *n* = 6). mRNA expression of (**C**) SOD, (**D**) GPx, (**E**) catalase, (**F**) GDNF, (**G**) BDNF, as quantified via qRT-PCR in primary glial cells. (**A**–**G**) Data represent the mean ± SD (*n* = 6). Significantly different are * *p* < 0.05, *** *p* < 0.001 compared to control; ^#^ *p* < 0.05, ^##^ *p* < 0.01 and ^###^ *p* < 0.001 compared to rotenone.

As shown in Figure 3A,B, the treatment of cells with rotenone led to a significant increase (2.7-fold) in intracellular ROS levels (*** *p* < 0.001, Figure 3B). In comparison, co-treatment with FCE (250, 500 μg/mL) remarkably reduced rotenone-induced ROS production by only 2.3 and 2.0 fold, respectively (FCE 250 μg/mL, ^###^ *p* < 0.001; FCE 500 μg/mL, ^###^ *p* < 0.001, compared to rotenone, Figure 3B). The results clearly disclosed that the FCE treatment attenuated the oxidative stress induced by rotenone.

### 3.6. FCE Halted Rotenone-Induced Decrease in mRNA Expression of Antioxidant Enzymes in Primary Glial Cells

Oxidative stress has been cited in a number of published reports as a cause of Parkinson’s disease [32]. Specifically, front-line defense antioxidants, involving superoxide dismutase (SOD), catalase (CAT) and glutathione peroxidase (GPX), are protective against scavenging toxic free radicals from the human body [33].

A qRT-PCR was used to determine the evolution of the mRNA expression of antioxidant enzymes in mouse primary glial cells following rotenone-induced oxidative stress and an FCE-mediated protection process.

The mRNA expression levels of antioxidant enzymes (SOD, GPx, catalase) were markedly declined in the primary glial cells with rotenone treatment, compared to the control group (*** *p* < 0.001, Figure 3C,D,E, respectively).

In comparison with the rotenone group, the rotenone-challenged cells treated with 250 and 500 μg/mL FCE exhibited augmented mRNA expression levels in SOD (^###^ *p* < 0.001, FCE 500 μg/mL, Figure 3C), GPx (^##^ *p* < 0.01, FCE 500 μg/mL, Figure 3D) and catalase (^###^ *p* < 0.001, FCE 500 μg/mL, Figure 3E). The results revealed that FCE treatment significantly attenuated the rotenone-induced decrease in mRNA expression of antioxidant enzymes.

### 3.7. FCE Augmented mRNA Expression of Neurotrophic Factors in Primary Glial Cells

The mRNA expression was then measured using qRT-PCR. Compared with the control group, when the primary glial cells were treated with rotenone, the mRNA expression levels of GDNF and BDNF were decreased significantly (*** *p* < 0.001, Figure 3F; *** *p* < 0.001, Figure 3G, respectively). In comparison, when rotenone was co-treated with FCE (250, 500 μg/mL), the mRNA expression levels of the neurotrophic factors were significantly increased, compared with the rotenone group (GDNF, ^###^ *p* < 0.001, FCE 250 μg/mL, ^###^ *p* < 0.001, FCE 500 μg/mL, Figure 3F; BDNF, ^###^ *p* < 0.001, FCE 250 μg/mL, ^###^ *p* < 0.001, FCE 500 μg/mL, Figure 3G, respectively). 

These results showed that FCE attenuated the rotenone-induced downregulation of mRNA levels of neurotrophic factors in glial cells, thereby enhancing the benefits of glial support and the ultimate neuronal survival potential during glial-neuronal cross-talk.

### 3.8. FCE Protected against Rotenone-Induced Apoptosis in Primary Glial Cells 

The effect of FCE upon rotenone-induced apoptosis in the primary glial cells was determined through sub-G1 assay and Annexin V/propidium iodide assay. Four panels of cells comprising control, rotenone, and rotenone + 250 and 500 μg/mL FCE were stained with the DNA-binding dyes, Annexin V/FITC and propidium iodide. 

**Figure 4:**
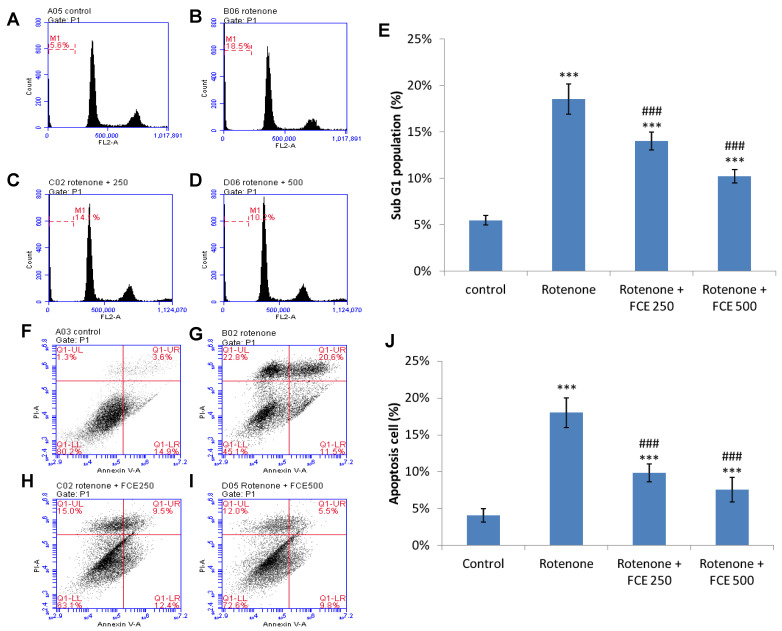
FCE prevented rotenone-triggered apoptosis in primary glial cells. The extent of apoptosis was determined as positive cells for subG1 phase (**A**–**E**) and annexin V-fluorescein isothiocyanate/propidium iodide method (**F**–**J**) in flow cytometric analysis. Primary glial cells were treated with vehicle or rotenone 200 μM and FCE (0, 250, 500 μg/mL) for 24 h. Flow cytometry was conducted to investigate the following groups. (i) Control group (**A**,**F**); (ii) treatment with 200 μM rotenone (**B**,**G**); (iii) treatment with rotenone and 250 μg/mL FCE (**C**,**H**); (iv) treatment with rotenone and 500 μg/mL FCE (**D**,**I**). Results for the proportional (%) of sub-G1 phase population and the apoptotic cells (%) positive for annexin V staining in each group are shown in E, and J, respectively. (**A**–**J**) Data represent the mean ± SD (*n* = 6). Significantly different are *** *p* < 0.001 compared to control; ^###^ *p* < 0.001 compared to rotenone.

As indicated by flow cytometric assessment, the cells treated with rotenone exhibited a greater level of sub-G1 phase. The cells treated with 200 μM rotenone showed 18.54% in the sub-g1 phase (Figure 4B,E) when compared to the basal level of control cells at 5.49% (*** *p* < 0.001, Figure 4A,E). In comparison with the rotenone-treated cells, the rotenone-challenged cells treated with FCE significantly depressed the larger proportion of rotavirus-induced sub-G1 phase, down to 14.02% (FCE 250 μg/mL, ^###^ *p* < 0.001, Figure 4C,E) and 10.24% (FCE 500 μg/mL, ^###^ *p* < 0.001, Figure 4D,E), respectively.

Accordingly, the apoptosis ratios in the 200 μM rotenone-treated cells and control cells were 18.00 % and 4.07 %, respectively (*** *p* < 0.001, Figure 4J). The cells treated with rotenone and co-conditioned with FCE markedly diminished rotenone-induced apoptosis, significantly reducing it to 9.86% (FCE 250 μg/mL, ^###^ *p* < 0.001, Figure 4J) and 7.57 % (FCE 500 μg/mL, ^###^ *p* < 0.001, Figure 4J), respectively. 

Taken together, these findings imply that FCE offers therapeutic potential for reducing injury-triggered neuroinflammation and ROS formation. Moreover, FCE was shown to potentiate attenuated benefits from injury, at least at the transcriptional level, including the production of antioxidant enzyme and BDNF in glial cells, a pivotal contributor to neurodegenerative processes. Through these benefits, glial cells were accordingly protected from injury-induced apoptosis, thus restoring the protective function of supporting neuronal survival and microenvironmental homeostasis. 

## 4. Discussion

Because of the adverse effects of synthetic pharmaceuticals, the World Health Organization estimates that more than 80% of the population, as in African and Asian countries, prefer traditional strategies to chemical drugs [34]. For example, herbal drugs, such as curcumin, aspirin, quinine, and triphala, were widely found in many modern medicinal formulations [34,35]. To characterize the pharmacological profile of FCE as a complementary drug to reduce inflammation or increase the neuroprotective effect of glial cells is necessary for the management of CNS diseases.

The overall protective effects of FCE supplementation can improve endurance and reduce exercise-induced muscle damage, inflammatory stress and liver damage [36]. In an in vitro model, pretreatment with FCE reduced ischemia-reperfusion injury in hepatocytes and inhibited the release of the pro-inflammatory cytokine TNF-α, while increasing the anti-inflammatory IL-10 cytokine [37]. 

In animal models of inflammation and oxidative stress upon the liver, FCE significantly reduced the hepatic inflammation (indexed levels of aspartate aminotransferase (AST) and alanine aminotransferase (ALT)), decreased the thiobarbituric acid reactive substances (TBARS) and reduced hydroxyproline collagen synthase in the livers of carbon tetrachloride (CCl4)-treated rats [18]. FCE attenuated the hepatic oxidative stress induced by monosodium glutamate, as evidenced by reduced serum amino transaminase activity, alkaline phosphatase, lipid parameters, hepatic malondialdehyde levels (MDA), and NO and increased the activity of antioxidant enzymes in the liver, such as SOD and catalase, as well as reducing glutathione (GSH) [38]. 

In addition to its hepatic protective effects, FCE has been shown to exert a systemic anti-inflammatory effect. FCE has been found to inhibit pro-inflammatory TNF-α release and to reduce the levels of AST, ALT and lactic dehydrogenase (LDH) after hemorrhagic shock in rats [39]. This critical protective effect deserves to be confirmed in the CNS, as proposed in the current study. 

Neuroinflammation, as a constituent of progressive neurodegenerative processes, has been increasingly demonstrated to be implicated in the pathogenesis of PD. Injury-stimulated microglia activation and its subsequent astrocyte reactivation [40], as markers of neuroinflammation, have been frequently assayed in PD patients and experimental animal models. It is well known that microglia-mediated neurotoxicity is a crucial molecular mechanism associated with cell death, damage and functional deterioration of neurons seen in several neurodegenerative diseases including PD. Glia-activation-mediated neurotoxicity is known to be a key molecular mechanism linked to cell death, injury and neuronal functional degeneration in an array of neurodegenerative diseases, including PD [41,42,43]. Specifically, in the presence of excessive levels of extracellular stimulation, microglia are most commonly activated by stress and in turn secrete large amounts of pro-inflammatory cytokines, including TNF-α and IL-1β, which play an important role in the etiology of PD [41]. Injury-stimulated inflamed glial cells can exacerbate neurodegenerative diseases by producing and releasing neurotoxins, such as ROS and NO [44]. NO has been shown to be engaged in the degeneration of oligodendrocytes in multiple sclerosis and the eventual neuronal death in AD and PD [45]. In the present study, using the model of inflammation in primary glial cells, FCE was shown to significantly inhibit the expression of the pro-inflammatory cytokines iNOS, TNF-α and IL-1β mRNA in primary glial cells and suppress LPS-stimulated NO release and production of the pro-inflammatory cytokine TNF-α. In the pathogenesis of neurodegenerative diseases, the suppression of the initial inflammatory response offers the potential to prevent succeeding inflammation-induced mitochondrial dysfunction and, ultimately, neuronal apoptosis.

Under normal circumstances, the level of free radicals can be modulated through the cellular antioxidant protective mechanism to restore cellular homeostasis [46,47]. Oxidative stress has been evidenced to increase the risk of neurodegenerative diseases, including PD [48,49]. It has been shown that rotenone administration can immediately increase ROS production and lead to damage to dopaminergic neurons [25,28,48,49,50]. In the present study, rotenone-induced cytotoxicity, as implicated in PD, was conducted in an in vitro PD model. The results indicated that rotenone stimulation induced a significant increase in ROS levels, reduced the mRNA expression of SOD, catalase and GPx, led to apoptosis and increased sub-G1 phase levels. FCE treatment was effective at reducing elevated ROS values. Taken together, our data suggested that FCE attenuated the generation of inflammatory responses under injury and also attenuated the oxidative damage induced by rotenone, which illustrates the therapeutic potential of FCE application in neurodegenerative diseases to alleviate two major etiologies: inflammation and mitochondrial dysfunction.

An alternative and complementary approach to treating this neurological disorder is to use neurotrophic factors to reduce progressive neuronal loss [51,52,53]. Specific neurotrophic factors, such as GDNF [51,53,54,55] and BDNF [56,57,58], have been demonstrated to attenuate the lesion-induced loss of nigrostriatal dopaminergic neurons in animal models of PD. Notably, GDNF and BDNF can interfere with apoptotic and necrotic forms of cell death. GDNF is a valid survival factor for injured nigrostriatal dopaminergic neurons and is being assessed as a potential treatment for PD [51]. Research in animal models of PD with intracerebral injection of recombinant GDNF protein has shown that GDNF can effectively protect injured nigrostriatal neurons and stimulate dopamine turnover/release among rescued neurons [59,60]. The delivery of GDNF into the brain via a vector has been shown to protect nigrostriatal neurons in rodent [51,55] and monkey [53,61] PD models. 

A similar protective effect on nigrostriatal dopaminergic neurons, in addition to GDNF, has been investigated in BDNF [56,57]. In a PD animal model, the transplantation of modified BDNF-expressing fibroblasts into the striatum or midbrain can mitigate 6-hydroxydopamine (6-OHDA)-induced loss of nigrostriatal neurons [62,63]. In addition, animal data showed that the enhanced expression of BDNF in striatal cells via AAV vectors could enhance functional recovery from 6-OHDA lesions. [58]. Dopaminergic neurotransmission in nigrostriatal neurons can be modulated by BDNF, as shown in the heightened rotational behavior and the increased turnover of dopamine in the striatum [57]. In the present study, using an in vitro glial cell model of rotenone-induced neurotoxicity, FCE was shown to attenuate the decline in GDNF/BDNF production upon rotenone-stimulated primary glial cells. The results from the experimental data confirmed that apart from its typical anti-inflammatory and protective effects against oxidative stress, FCE exerted complementary benefits in preventing the decline of neurotrophic factors, which help to combat harmful pathogenic factors in the progression of PD. 

There are several experimental limitations that need to be addressed. First, although exerting anti-inflammatory, antioxidant and anti-aging effects, the exact components of FCE, as a form of extract, are not likely to be precisely determined for the respective properties. According to our previous data, from gas chromatography–mass spectrometry [20], the functional compounds of FCE are campesterol (C28H48O, 5.5%), dousterol (C29H48O, 3.2%) (belonging to steroids), cis-9-octadecenoic acid (C18H34O2, 5.8%) and Z-11-hexadecenoic acid (C16H30O2, 3.4%) (belonging to fatty acids), which all exhibited a protective effect against pro-inflammation [64,65,66,67]. A more extensive investigation is needed to further explore the most effective neuroprotective component of FCE for more detailed protective molecular signaling in the perspective of FCE-based therapeutic strategies. Moreover, based on the results of our study, FCE showed positive anti-inflammatory and anti-apoptotic effects, as well as enhancing the expression of BDNF/GDNF mRNA. These beneficial effects could explain the protective effect of FCE on glial cell survival. However, as shown in Figure 1A, glial cell cultures incubated with FCE alone presented cell survival rates higher than those of control cells. Therefore, the exact protective effect of FCE on cell survival or enhanced cell proliferation cannot be definitely distinguished in this setting. Further mechanistic studies are warranted. 

In addition, as mentioned earlier, glial cells, including microglia, astrocytes and oligodendrocytes, jointly play a role in the pathological mechanisms of neurodegenerative diseases, such as PD [41,42,43]. With excessive immunocompetence during aging or abnormal protein folding/aggregation caused by environmental and genetic factors, microglia activation remains the initial step towards the neuroinflammatory response. Stimulated M1-phase microglia have been shown to directly activate astrocytes and reciprocally modulate the innate immune defenses of the CNS (via microglia-astrocyte crosstalk) [68]. Inflamed glial cells further exacerbate the neurodegenerative process by producing and releasing neurotoxins such as NO [44], which is involved in the degeneration of oligodendrocytes in multiple sclerosis and in the death of neurons in AD and PD [45]. Since immune and inflammatory responses in the CNS are mainly coordinated by the interaction between glial cells and neurons in the brain, the current study used cultures of microglia, astrocytes and oligodendrocytes, i.e., mixed glial cell cultures, which were conducted to mimic the microenvironment in which glial cells coexist on the pathological basis of neurodegeneration. The rationale for not isolating individual glial cells in the current study was mainly to apply mixed primary glial cells to simulate the circumstances of a real brain and to observe whether FCE was effective in mitigating neuroinflammatory responses on an in vitro model of glial cell injury. However, the respective roles of microglia or astrocytes involved in FCE-mediated protection, including the phenotypic changes of astrocytes and microglia after FCE treatment in the presence of LPS/rotenone could not be completely analyzed. Further cell-specific experiments were considered to clarify the role played by individual glial cells in the protective effect of FCE. Finally, attempts to simulate glial cell reactivation in the microenvironment, neuroinflammation or ROS formation from an insult to the LPS or rotenone in primary glial cell cultures are unlikely to achieve an approximately equivalent pathology implicated in neurodegenerative diseases, including PD. However, from the data we presented, both in our previous publication on MPTP-induced PD animals [19] and injury-stimulated glial cells, FCE showed an excellent anti-inflammatory response, which is an important therapeutic landmark for use as pharmacological agents targeting neurological diseases. 

## 5. Conclusions 

In light of neuroinflammation and mitochondrial dysfunction upon glial cell reactivation linked to PD pathogenesis, our previous data from animal models of PD showed that FCE can attenuate injury-induced neuronal depletion. The present study confirms the mechanism of FCE protection in details, including anti-inflammation, anti-oxidative stress, the prevention of neurotrophic factor exhaustion and, eventually, anti-apoptosis in injury-stimulated glial cells. The results of these in vivo and in vitro studies endorse the therapeutic potential of FCE in the management of neurodegenerative diseases, including PD. 

## Figures and Tables

**Table 1:** Specific primers.

cDNA Target		Sequence (5’ -> 3’)	Product Size (bp)	Sequence Reference
Actin	forward	GCTACAGCTTCACCACCACA	123	NM_007393.5
	reverse	TCTCCAGGGAGGAAGAGGAT		
BDNF	forward	TGGCTGACACTTTTGAGCAC	131	NM_001316310.1
	reverse	CAAAGGCACTTGACTGCTGA		
GDNF	forward	TGGGCTATGAAACCAAGGAG	142	NM_001301357.1
	reverse	CAACATGCCTGGCCTACTTT		
TNF-α	forward	CAGGGGCCACCACGCTCTTC	371	NM_001278601.1
	reverse	CTTGGGGCAGGGGCTCTTGAC		
IL-1β	forward	CAGGCTCCGAGATGAACAACAAAA	332	NM_008361.4
	reverse	TGGGGAACTCTGCAGACTCAAACT		
iNOS	forward	TCACTGGGACAGCACAGAAT	510	NM_001313922.1
	reverse	TGTGTCTGCAGATGTGCTGA		
GPx	forward	CCTCAAGTACGTCCGGCCTG	197	NM_008160.6
	reverse	CAACATCGTTGCGACACACC		
SOD	forward	TGGGTTCCACGTCCATCAGTA	151	NM_011434.1
	reverse	ACCGTCCTTTCCAGCAGTCA		
Catalase	forward	TTCAGAAGAAAGCGGTCAAGAAT	59	NM_009804.2
	reverse	GATGCGGGCCCCATAGTC		

## Data Availability

The data used to support the findings of this study are included within the article.
